# Phenotypic and genotypic investigation of carbapenemases in carbapenem-resistant *Klebsiella pneumoniae* isolates

**DOI:** 10.1007/s12223-026-01443-z

**Published:** 2026-03-07

**Authors:** Serpil Genç, Ayten Nur Uzun, Hilal Tanriverdi̇

**Affiliations:** Faculty of Medicine, Department of Medical Microbiology, Kütahya Health Sciences University, Kütahya, Turkey

**Keywords:** OXA-48, NDM, KPC, IMP, VIM

## Abstract

Carbapenem resistance in *Klebsiella pneumoniae* is primarily mediated by carbapenemase production. This study aimed to identify carbapenemase genes in carbapenem-resistant *K. pneumoniae* isolates using multiplex polymerase chain reaction (PCR) and to evaluate the performance of the combination disk assay (CDA) as a phenotypic detection method. A total of 123 carbapenem-resistant *K. pneumoniae* isolates were analyzed. Colistin and meropenem susceptibility were determined by broth microdilution, while ceftazidime-avibactam susceptibility was assessed using disk diffusion. Five common carbapenemase genes (*blaOXA-48*, *blaNDM*, *blaKPC*, *blaIMP*, *blaVIM*) were detected by multiplex PCR. CDA was performed using meropenem and temocillin disks in combination with specific inhibitors (EDTA, boronic acid, cloxacillin). Colistin (59.3%) and ceftazidime-avibactam (58.5%) showed the highest susceptibility rates. PCR identified *blaOXA-48* in 53.6%, *blaKPC* in 45.5%, and *blaNDM* in 32.5% of isolates. CDA detected MBL, KPC, and OXA-48 enzymes in 44.7%, 44.7%, and 10.6% of isolates, respectively, and failed to detect OXA-48 in the presence of NDM. Compared with PCR, CDA showed excellent agreement for KPC detection (sensitivity 100%, specificity 100%; κ = 0.984), moderate agreement for MBL (sensitivity 95%, specificity 76.1%; κ = 0.679), and poor agreement for OXA-48 (sensitivity 19.7%, specificity 100%; κ = 0.185). CDA may be used as an initial phenotypic screening tool for the detection of KPC-producing isolates; however, its limited ability to detect OXA-48 and carbapenemase co-production restricts its reliability for broader clinical interpretation. Therefore, PCR-based methods remain essential for accurate confirmation and surveillance.

**Supplementary Information:**

The online version contains supplementary material available at https://doi.org/10.1007/s12223-026-01443-z.

## Introduction

According to the 2023 European Antimicrobial Resistance Surveillance report, Türkiye has shown a progressive increase in carbapenem resistance among *Klebsiella pneumoniae* isolates over the years, with rates of 39.4%, 48.2%, and 49.1% reported in 2019, 2020, and 2021, respectively (World, 2023). In the 2022 national surveillance report, this rate further escalated to 63.6% (Türkiye Halk Sağlığı Genel Müdürlüğü 2022).

Carbapenem resistance in *Enterobacterales* is primarily due to the production of carbapenemases—β-lactamases that hydrolyze carbapenems. While OXA-48 remains the most prevalent carbapenemase type in *Enterobacterales* in Türkiye, other enzymes such as VIM, IMP, NDM, and KPC have also been documented in multiple national studies (Gülay 2014; Us et al., 2010; Eser et al. 2014; Genç et al. 2022).

Phenotypic confirmation methods for carbapenemase detection, as recommended by the European Committee on Antimicrobial Susceptibility Testing (EUCAST) (2017), include combination disk assays, colorimetric tests, carbapenem hydrolysis methods, and lateral flow immunoassays. In CDA, boronic acid is recommended for the detection of KPC, while EDTA (ethylenediaminetetraacetic acid) or dipicolinic acid is used for metallo-β-lactamase (MBL) detection, including NDM. Currently, no inhibitor is available for class D enzymes such as OXA-48. In the absence of synergy with KPC or MBL inhibitors, high-level temocillin resistance (MIC > 32 mg/L or inhibition zone < 11 mm with a 30 µg disk) is a phenotypic indicator of OXA-48 production. A cloxacillin disk is used to detect AmpC hyperproduction and porin loss (EUCAST 2017).

This study aimed to identify carbapenemase genes in carbapenem-resistant *K. pneumoniae* isolates using multiplex polymerase chain reaction (PCR) and to evaluate the diagnostic performance of CDA as a cost-effective phenotypic method. By comparing the genotypic and phenotypic profiles of carbapenemase production, this study provides epidemiological insights and assesses the utility of CDA for routine laboratory implementation under resource-limited conditions.

In addition, the observed emergence of KPC producing isolates may reflect a shift in enzyme distribution patterns in Türkiye, warranting closer epidemiological monitoring.

## Materials and methods

### Bacterial isolates and antimicrobial susceptibility testing

A total of 123 non-duplicate *K. pneumoniae* isolates, recovered from various clinical specimens between October 2023 and July 2024, were included in this study. Bacterial identification and antimicrobial susceptibility testing were performed using the Phoenix system (BD), Autof MS 2600, and AutoMic-i600 (Autobio, China). All isolates were confirmed as carbapenem-resistant according to EUCAST (European Committee on Antimicrobial Susceptibility Testing 2024) breakpoints.

Isolates were stored in skimmed milk medium at − 80 °C until testing. Minimum inhibitory concentrations (MICs) for meropenem and colistin were determined by the broth microdilution method using cation adjusted Mueller–Hinton broth, in accordance with EUCAST guidelines. Stock solutions of antimicrobial agents were prepared in sterile distilled water and stored at − 80 °C. *Escherichia coli* ATCC 25,922 was used as the quality control strain. Ceftazidime-avibactam (CZA) susceptibility was determined using the disk diffusion method and interpreted based on EUCAST 2024 criteria.

### Investigation of carbapenemase presence

Five carbapenemase genes (*blaOXA-48*,* blaNDM*,* blaIMP*,* blaVIM*,* blaKPC*) which are among the most common genes responsible for carbapenem resistance in Türkiye, were investigated genotypically by multiplex PCR and phenotypically CDA. The CDA method used antibiotic disks containing meropenem and temocillin together with meropenem disks impregnated with inhibitors (EDTA, Boronic Acid (BA), cloxacillin).

#### Genotypic method: PCR

Total DNA was extracted using the boiling method (Steinbrueckner et al. 1999). Multiplex PCR using previously described primers was performed to detect the presence of carbapenemase genes (*blaOXA-48*,* blaNDM*,* blaIMP*,* blaVIM*,* blaKPC*) (Genç et al. 2022).

Multiplex PCR for carbapenemase genes were performed in two different multiplex reactions, with the mixture and thermal cycling conditions as previously described; *blaIMP* and *blaVIM* were detected in the first reaction, and *blaOXA-48*, *blaNDM* and *blaKPC* were detected in the second reaction (Genç et al. 2022). Positive controls for all targeted genes, including VIM and IMP, were included in each PCR assay.

PCR products were analyzed on a 2% agarose gel, run at 110 V for 50 min, and visualized under UV light.

#### Phenotypic method: CDA

A 0.5 McFarland inoculum of each bacterium was prepared and spread on Mueller–Hinton agar plates. Five disks (Bioanalyse, Ankara, Türkiye) were placed on each plate: meropenem 10 µg, meropenem 10 ug+ boronic acid 600 ug (MEM BA), meropenem 10 ug+ cloxacillin 750 ug (MEX), meropenem 10 ug+ EDTA 292 ug (MEL) and temocillin 30 ug. After overnight incubation, an increase of > 5 mm in the zone diameter around the inhibitor containing disks compared to the meropenem only disks was considered as a positive result for EDTA and cloxacillin. A > 4 mm increase was accepted as a positive result for BA (European Committee on Antimicrobial Susceptibility Testing 2017; Us et al. 2016). Synergy with EDTA was interpreted as MBL production, synergy BA as KPC production, and synergy with cloxacillin as AmpC production (Kılıç et al. 2016; Zhang et al. 2022).

In the absence of synergy with any inhibitor, high-level temocillin resistance (zone diameter < 11 mm) was considered indicative of OXA-48 production (European Committee on Antimicrobial Susceptibility Testing 2017). Well-characterized KPC- and MBL-producing clinical isolates were used as positive controls for CDA validation.

## Statistical analysis

Statistical analysis was performed using IBM SPSS 30.0 (SPSS Inc., Chicago, IL, USA). The agreement between multiplex PCR and the inhibition-based CDA method for detecting carbapenemase genes was evaluated using the kappa test, while statistical relationships were assessed using Fisher’s exact test. The chi-square test was used to compare carbapenemase types with ceftazidime-avibactam susceptibility. A p-value < 0.05 was considered statistically significant.

## Results

### Bacterial isolates and antimicrobial susceptibility testing

Among the 123 carbapenem-resistant *K. pneumoniae* isolates, 85 (69.1%) were obtained from ICU patients, 34 (27.6%) from general wards, and 4 (3.3%) from outpatients. Isolates originated from respiratory tract (*n* = 47), blood-catheter (*n* = 41), urine (*n* = 26), wounds (*n* = 8), and one abscess.

Susceptibility profiles against 15 antimicrobial agents are presented in Table 1. Colistin (59.3%) and CZA (58.5%) were the most effective agents.

**Table 1:** Antimicrobial susceptibility profiles of isolates (*n* = 123)

Antibiotic	Susceptible (S)	Resistant (*R*)	Intermediate (I)
KOL	59.3%	40.7%	-
CZA	58.5%	41.5%	-
AK, CN	48%	52%	-
SXT	13.8%	84.6%	1.6%
MEM, IMP, ETP	0%	100%	-
TZP, AMC, CRO, CAZ, FEP	0%	100%	-
CIP, LEV	0%	100%	-

Data are presented as percentages of isolates categorized as susceptible (S), resistant (R), or intermediate (I) according to EUCAST 2024 guidelines

*AMC* amoxicillin-clavulanic acid, *TZP* piperacillin-tazobactam, *SXT* trimethoprim-sulfamethoxazole, *CAZ* ceftazidime, *CIP* ciprofloxacin, *CN* gentamicin, *AK* amikacin, *ETP* ertapenem, *IMP* imipenem, *MEM* meropenem, *KOL* colistin, *CZA* ceftazidime-avibactam, *FEP* cefepime, *LEV* levofloxacin, *CRO* ceftriaxone

### PCR

**Fig. 1:**
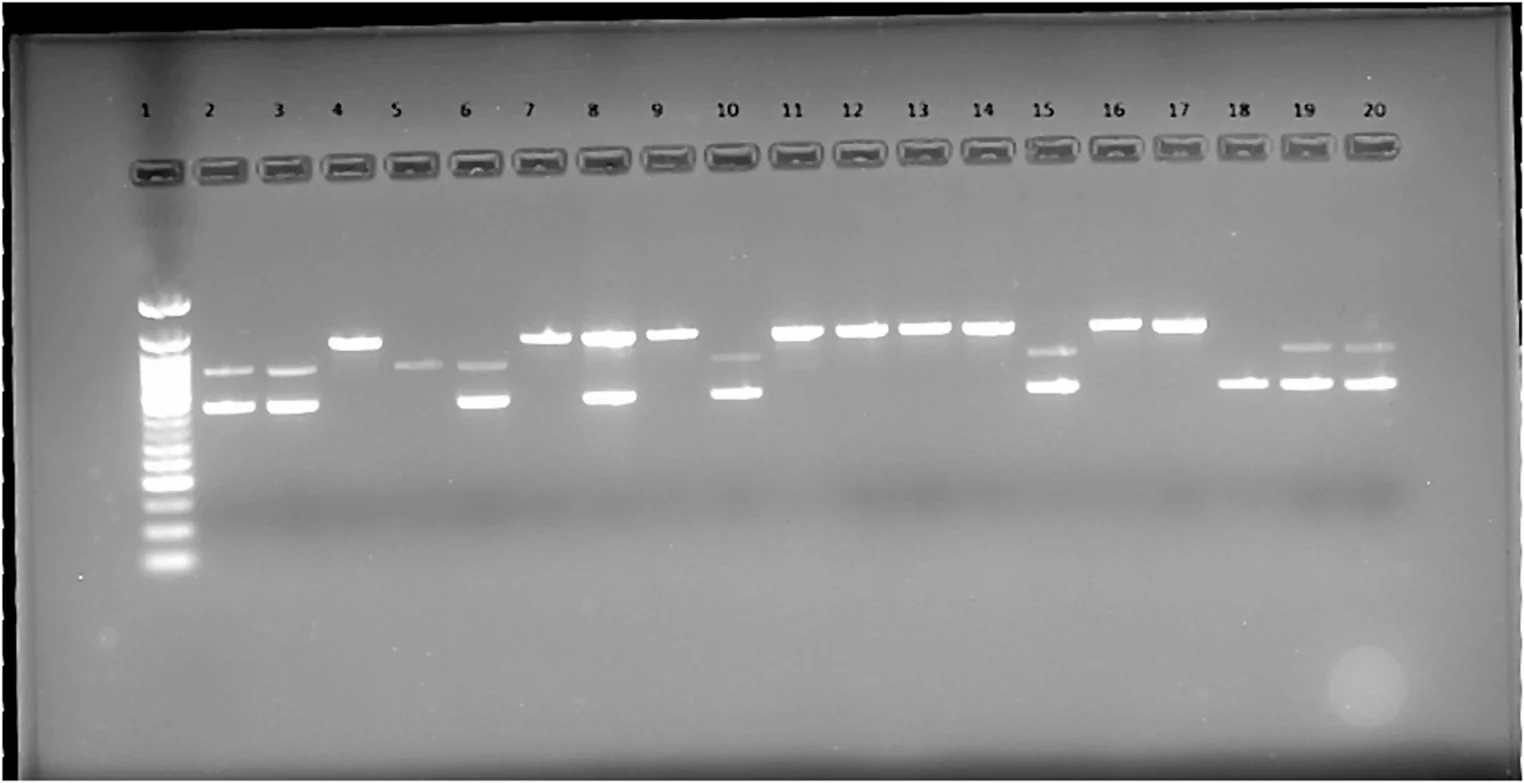
Representative gel electrophoresis image showing PCR products for carbapenemase genes in *Klebsiella pneumoniae* isolates. Lane 1: 50 bp DNA marker; Lanes 2, 3, 6, 10, 15, 19, 20: co-producers of *blaOXA-48* and *blaNDM*; Lane 5: *blaNDM*-positive isolate; Lane 4, 7, 9, 11–14, 16, 17: *blaKPC*-positive isolates; Lane 8: *blaKPC + OXA-48* co-producer; Lane 18: *blaOXA-48*-positive isolate. Bands were visualized under UV light after electrophoresis on 2% agarose gel at 110 V for 50 min

*blaIMP* and *blaVIM* were not detected in any isolates. The distribution of carbapenemase genes detected by PCR is shown in Table 2. A representative gel image is provided in Fig. 1.

**Table 2:** Distribution of carbapenemase genes detected by PCR

Gene	No. of Isolates (*n*), (%)
*blaKPC*	52 (42.3%)
*blaOXA-48 + NDM*	35 (28.5%)
*blaOXA-48*	29 (23.6%)
*blaKPC + NDM*	2 (1.6%)
*blaKPC + OXA-48*	2 (1.6%)
*blaNDM*	3 (2.4%)
Total	123

### CDA

**Fig. 2:**
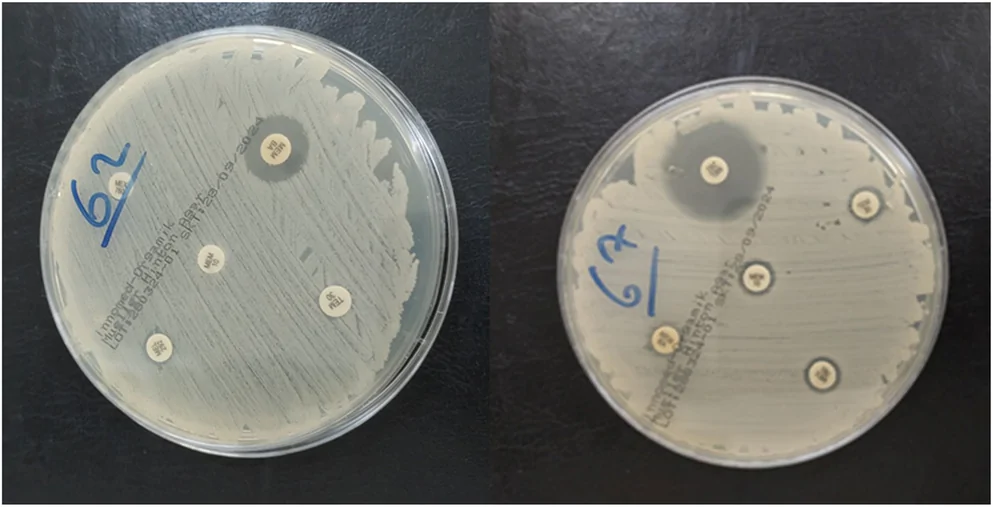
Phenotypic detection of carbapenemase production using the combination disk assay (CDA). Left panel: Class A (KPC) producer showing ≥ 5 mm increase in zone diameter with boronic acid-impregnated meropenem disk compared to meropenem alone Right panel: Class B (MBL) producer showing synergy with EDTA-impregnated disk Disks used: MEM = meropenem (10 µg), MEM-BA = meropenem + boronic acid (600 µg), MEL = meropenem + EDTA (292 µg), MEX = meropenem + cloxacillin (750 µg), TEM = temocillin (30 µg)

The distribution of carbapenemase enzymes detected by CDA is shown in Table 3, and a sample plate image of the test is provided in Fig. 2.

**Table 3:** ***Distribution of carbapenemase enzyme classes detected by CDA***

Enzyme	No. of Isolates (*n*), (%)
Class B (MBL)	55 (44.7%)
Class A (KPC)	55 (44.7%)
Class D (OXA-48)	13 (10.6%)
Total	123

### Comparison of PCR and CDA results

By PCR, 123 *K. pneumoniae* isolates carried at least one carbapenemase gene. The detected frequencies were 53.6% for *blaOXA-48*, 45.5% for *blaKPC*, and 32.5% for *blaNDM*. The co-occurrence rates of *blaOXA-48 + NDM*, *blaNDM + KPC*, and *blaOXA-48 + KPC* were 28.5%, 1.6%, and 1.6%, respectively.

In CDA, class B (MBL), class A (KPC), and class D (OXA-48) enzymes were detected at rates of 44.7%, 44.7%, and 10.6%, respectively. All isolates identified as *blaOXA-48 + NDM* by PCR were interpreted as class B by CDA, resulting in missed detection of class D enzymes. Similarly, 17 isolates carrying blaOXA-48 alone were misclassified as class B (Table 4). Concordance analysis demonstrated excellent agreement between CDA and PCR for KPC (κ = 0.984), good agreement for MBL (κ = 0.679), and very poor agreement for OXA-48 (κ = 0.185) (Table 5). While CDA showed high sensitivity and specificity for KPC detection, and high sensitivity but an increased false-positive rate for MBL, its performance for OXA-48 was limited by very low sensitivity despite perfect specificity. This limitation was particularly evident in isolates co-producing two different carbapenemases, where CDA consistently identified only a single enzyme type, contributing to reduced concordance and lower κ values.

**Table 4:** Comparison of carbapenemase gene detection by PCR and phenotypic classification by CDA

PCR Result	CDA Result	No. of Isolates (*n*)	Expected Enzyme(s)	Misclassification
*blaKPC*	Class A (KPC)	52	KPC	No
*blaKPC + OXA-48*	Class A (KPC)	1	KPC + OXA-48	Yes (OXA-48 missed)
*blaKPC + NDM*	Class A (KPC)	2	KPC + NDM	Yes (NDM missed)
*blaOXA-48 + NDM*	Class B (NDM)	35	OXA-48 + NDM	Yes (OXA-48 missed)
*blaOXA-48 + NDM*	Class D (OXA-48)	1	OXA-48 + NDM	Yes (NDM missed)
*blaOXA-48*	Class D (OXA-48)	12	OXA-48	No
*blaOXA-48*	Class B (NDM)	17	OXA-48	Yes (OXA-48 misclassified as MBL)
*blaNDM*	Class B (NDM)	3	NDM	No

Misclassification indicates a discrepancy between PCR and CDA results, especially in co-producing isolates

**Table 5:** Agreement between PCR and CDA for detecting carbapenemase classes

CDA	kappa	PPV	NPV	Sensitivity	Specificity
Class D (OXA-48)	0.185 (poor)	100%	51.81%	19.69%	100%
Class A (KPC)	0.984 (excellent)	100%	98.52%	100%	100%
Class B (MBL)	0.679 (good)	69.09%	97.05%	95%	76.1%

Diagnostic performance was assessed using kappa statistics, sensitivity, specificity, positive predictive value (PPV), and negative predictive value (NPV)

KPC-producing isolates were recovered from multiple hospital wards over the study period, without an obvious temporal or ward-based clustering (Supplementary Table S1).

### Comparison of carbapenemase genes with ceftazidime-avibactam and colistin susceptibility

When CZA susceptibility was evaluated according to carbapenemase genotype, isolates harboring *blaKPC* showed significantly higher susceptibility rates to CZA compared with isolates carrying non-KPC carbapenemase genes (*p* < 0.001).

Among isolates producing only *blaKPC*, 90.6% were susceptible to CZA. However, susceptibility decreased markedly when *blaKPC* was co-expressed with *blaNDM* or *blaOXA-48*. Due to the limited number of such co-producing isolates, statistical comparison between these profiles could not be performed.

Similarly, *blaOXA-48* alone was significantly associated with CZA susceptibility (*p* < 0.001), with a susceptibility rate of 46.4%. This rate dropped sharply to 2.9% when *blaNDM* was also present. Although susceptibility increased in isolates co-producing *blaKPC* and *blaOXA-48*, the number of such isolates was insufficient to assess statistical significance.

The presence of *blaNDM* was strongly associated with CZA resistance (*p* < 0.001). None of the isolates harboring *blaNDM* alone were susceptible to CZA. In contrast, zones interpreted as susceptible to CZA were observed in 22.9% of *blaOXA-48* + *blaNDM* producers and in 50% of *blaKPC* + *blaNDM* producers; however, these findings should be interpreted with caution given the known lack of avibactam activity against metallo-β-lactamases and the small number of isolates.

Colistin susceptibility rates did not differ significantly among carbapenemase gene profiles (*p* > 0.05).

### Meropenem MIC distributions

Meropenem MIC distributions, MIC₅₀ and MIC₉₀ values according to carbapenemase genotypes are shown in Table 6. MIC₅₀ values for all enzyme types were 512 µg/ml. Higher meropenem MIC values were observed in isolates harboring *blaNDM*, particularly when *blaNDM* was co-expressed with *blaOXA-48* or *blaKPC*. The lowest meropenem MIC values were 16 µg/ml, 128 µg/ml, and 64 µg/ml in isolates producing *blaOXA-48* alone, *blaNDM* alone, and *blaOXA-48 + blaNDM*, respectively. Similarly, the lowest meropenem MIC value increased from 32 µg/ml in *blaKPC*-only producers to 256 µg/ml in *blaKPC* + *blaNDM* co-producers.

**Table 6:** Meropenem MIC distributions in relation to carbapenemase gene profiles

Carbapenemase gene	MIC_50_ (µg/ml)	MIC_90_ (µg/ml)	MIC range
*blaKPC*	512	1024	32–2048
*blaOXA-48 + NDM*	512	1024	64–2048
*blaOXA-48*	512	512	16–1024
*blaKPC + NDM*	-	-	256, 1024
*blaKPC + OXA-48*	-	-	32, 512
*blaNDM*	-	-	128, 512, 512

## Discussion

Carbapenem-resistant *K. pneumoniae* (CRKP) isolates pose significant challenges worldwide due to their difficulty in treatment and their potential for rapid spread in hospitals. In this study, 69.1% of CRKP isolates were from ICU patients, consistent with reports indicating frequent isolation of resistant gram-negative bacteria in ICU settings (World Health Organization 2023).

CRKP isolates often show high levels of resistance to various antibiotic groups (Nordmann et al. 2011). In this study, high-level resistance was detected to most antibiotics (Table 1), and the most sensitive antibiotics were colistin (59.3%) and ceftazidime-avibactam (58.5%).

It is well-established that CZA is effective against class A, C and D carbapenemases, however ineffective against class B MBLs (Sümer et al. 2024). As expected, CZA was ineffective against MBLs, particularly NDM. Its susceptibility dropped to 0% in NDM-only isolates, while partial activity was observed when NDM was co-expressed with KPC (50%) or OXA-48 (22.9%). Interestingly, despite the known inhibitory activity of avibactam against OXA-48, the susceptibility rate observed in isolates harboring *blaOXA-48* alone was lower than anticipated. This finding suggests that reduced CZA susceptibility in OXA-48-producing isolates may not be solely attributable to the presence of the enzyme itself. Additional resistance mechanisms, such as porin loss, increased efflux pump activity, or the presence of other β-lactamases not inhibited by avibactam, may contribute to this phenomenon. Furthermore, strain-specific factors and variations in enzyme expression levels could also influence the phenotypic response to CZA.

Together, these findings reinforce the known spectrum of CZA and emphasize the need for genotypic confirmation when selecting empirical therapy.

Our PCR results highlight a shifting resistance landscape in Türkiye. While OXA-48 remains the most frequently detected carbapenemase—consistent with previous national studies (Us et al. 2010; Eser et al. 2014; Genç et al. 2016)—a significant rise in *blaKPC* was observed in this study, marking a notable epidemiological transition.

KPC, first reported in Türkiye in 2014 (Labarca et al. 2014), had been rarely or never detected in earlier studies (Genc et al. 2022; Demir et al. 2015), including one conducted by our own laboratory in 2016, which identified no KPC producers (Genç et al. 2016). In contrast, *blaKPC* was detected in 45.5% of isolates in the present study, making it the second most prevalent enzyme.

This increase aligns with global trends where KPC is gaining ground in regions where it was previously uncommon (Alvisi et al. 2025). The shift may be driven by selective antibiotic pressure or inter-hospital transmission. Given the high transmissibility of KPC producing strains—particularly in intensive care settings—these findings serve as an important warning for infection control strategies. Rapid molecular detection and targeted isolation are essential to curb further spread. Additionally, accurate identification of carbapenemase types remains critical for guiding appropriate antimicrobial therapy and limiting resistance development.

The high prevalence of KPC-producing isolates observed in this single-center study raises concern for possible dissemination. However, available ward distribution and temporal data did not demonstrate a clear clustering suggestive of a single localized outbreak (Supplementary Table S1). Instead, KPC-positive isolates were identified across multiple hospital units over the study period. Nevertheless, the absence of molecular typing methods such as Multilocus Sequence Typing (MLST) or Pulsed Field Gel Electrophoresis remains a limitation in definitively distinguishing clonal spread from a broader epidemiological shift.

The first report of NDM in Türkiye was made in 2012 (Poirel et al. 2012a), and subsequent notifications have increased in frequency (Çakar et al. 2016; Poirel et al. 2014). In this study, the third most common carbapenemase gene was *blaNDM*, detected at a rate of 38.9%. Given the significantly higher prevalence of NDM compared to previous national data, it can be predicted that NDM will become endemic in Türkiye in the coming years.

**Fig. 3:**
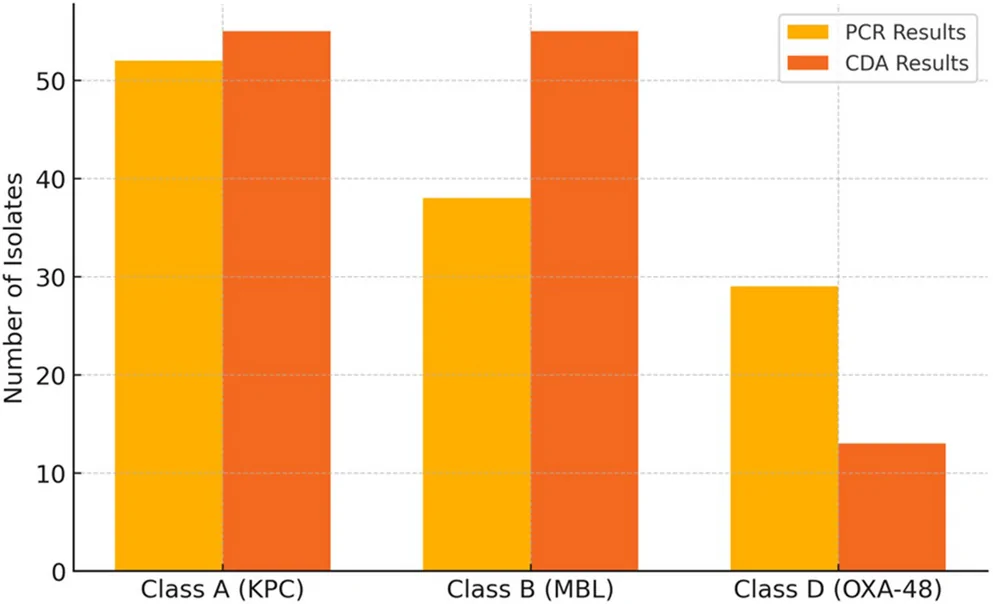
Comparison of PCR and CDA results for detection of carbapenemase classes in *K. pneumoniae* isolates. Bars indicate the number of isolates in which class A (KPC), class B (MBL), and class D (OXA-48) enzymes were detected by each method. CDA underestimated OXA-48 detection compared to PCR

CDA detected class B (MBL) and class A (KPC) enzymes at equal rates of 44.7%, whereas class D (OXA-48) was identified in only 10.6% of the isolates. However, it frequently failed to detect co-producing isolates, particularly those carrying both *blaOXA-48* and *blaNDM*, often misclassifying them as MBL producers and entirely missing the presence of OXA-48 (Fig. 3; Table 4). Seventeen *blaOXA-48* positive isolates confirmed by PCR were misinterpreted as MBL by CDA, highlighting this diagnostic limitation.

Although misclassification of OXA-48 as MBL may not critically affect routine diagnostics, since MBLs are associated with high level carbapenem resistance and share similar transfer dynamics, it poses a significant limitation for epidemiological studies and therapeutic guidance. The inability of CDA to distinguish co-produced enzymes diminishes its utility, especially in settings where accurate enzyme differentiation is critical for infection control.

Importantly, this limitation has direct clinical implications. In cases where *blaKPC* and *blaNDM* are co-produced, CDA frequently classifies such isolates as KPC alone, which may falsely suggest susceptibility to CZA. While CZA is an effective therapeutic option for infections caused by KPC-producing isolates, it is ineffective in the presence of NDM. Accordingly, reliance on CDA results alone to guide antimicrobial therapy may increase the risk of inappropriate treatment selection and potential therapeutic failure, particularly in regions with a high prevalence of carbapenemase co-production. These findings highlight the necessity of molecular confirmation prior to therapeutic decision-making.

In terms of accuracy, CDA showed excellent concordance with PCR for detecting class A (KPC), good concordance for class B (MBL), but poor performance for class D (OXA-48), with a sensitivity of only 19.7%, despite 100% specificity (Table 5). Notably, there is currently no specific inhibitor for phenotypic detection of OXA-48, and no standard phenotypic test is recommended for its reliable identification (Poirel et al. 2012b). In this method, absence of synergy with inhibitors alongside temocillin resistance was interpreted as OXA-48 production, while any observed synergy excluded OXA-48.

For MBL, CDA was found to have high sensitivity however a false positive rate of approximately 30%. While it can generally be considered a reliable test for MBL, confirming positive results with PCR may be advisable. The performance of inhibitor-based combination tests has been evaluated in various studies, and they have been reported to exhibit high sensitivity and specificity for KPC and NDM, suggesting their safe use for detecting bacteria producing these two enzymes (Genc et al. 2016; Doyle et al. 2012).

Due to its superior sensitivity and specificity, PCR remains the gold standard for carbapenemase detection and is particularly indispensable for the accurate identification of OXA-48-producing and co-producing strains. Given the lack of an effective phenotypic method for OXA-48 detection and the frequent misclassification of complex resistance profiles by CDA, which can lead to inadequate infection control measures and delayed or inappropriate antimicrobial therapy, using PCR instead of CDA for class D detection is a safer and more accurate approach.

MIC analyses revealed that the presence of NDM was associated with elevated meropenem MIC values, consistent with existing literature (Nordmann et al. 2011). Isolates co-harboring NDM and other carbapenemases showed higher MIC_50_ and MIC_90_ levels than single-producer isolates, further supporting the clinical impact of these resistance combinations.

While high-resolution molecular methods such as MLST, Whole Genome Sequencing or Western blot could offer more detailed epidemiological and mechanistic insights, these techniques were not performed due to resource and equipment limitations. Instead, this study focused on evaluating a phenotypic method that is practical and applicable under resource-constrained settings. Nonetheless, the findings underscore the critical need to incorporate molecular diagnostics, particularly PCR, into routine workflows to ensure accurate resistance profiling and guide appropriate antimicrobial strategies.

## Conclusions

CDA is a simple and cost-effective phenotypic screening method for detecting carbapenemase-producing bacteria, particularly KPC producers; however, its performance varies depending on the enzyme type. CDA is unreliable for OXA-48 detection and may yield false-positive results for metallo-β-lactamases. Importantly, CDA cannot reliably identify co-producing isolates, such as *blaKPC* and *blaNDM*, which may falsely suggest susceptibility to ceftazidime-avibactam and increase the risk of inappropriate therapy. Therefore, CDA should be used only as an initial screening tool, while PCR remains essential for accurate confirmation, therapeutic decision-making, and epidemiological surveillance.

The increasing detection of *blaKPC* and *blaNDM* alongside endemic *blaOXA-48* underscores the need for continued molecular monitoring to support infection control and antimicrobial stewardship.

## Supplementary Information

Below is the link to the electronic supplementary material.

Supplementary Material 1 (docx 14.2 KB)

Supplementary figure 4 (PNG 1.52 MB)

High Resolution Image (tif 10.3 MB)

Supplementary figure 5 (PNG 1.59 MB)

High Resolution Image (tif 10.3 MB)

Supplementary Material 2 (JPG 1.01 MB)

Supplementary figure 6 (PNG 1.61 MB)

High Resolution Image (tif 10.3 MB)

Supplementary figure 7 (PNG 1.47 MB)

High Resolution Image (tif 10.3 MB)

Supplementary figure 8 (PNG 1.50 MB)

High Resolution Image (tif 10.3 MB)

Supplementary figure 9 (PNG 1.54 MB)

High Resolution Image (tif 10.3 MB)

Supplementary figure 10 (PNG 1.48 MB)

High Resolution Image (tif 14.0 MB)

## Data Availability

All data generated or analyzed during this study are included in this published article.
