# Supplementary material for: Phenotypic and genotypic investigation of carbapenemases in carbapenem-resistant Klebsiella pneumoniae isolates
Source: Folia Microbiol (Praha). 2026 Mar 7;71(4):839–46. doi: 10.1007/s12223-026-01443-z (PMC13541925; doi:10.1007/s12223-026-01443-z)
Supplement: Supplementary file 1 [file 12223_2026_1443_MOESM1_ESM.docx]

Supplementary Table S1. Ward distribution of KPC-producing *K. pneumoniae* isolates

| **Ward** | **Number of KPC-positive isolates** |
| --- | --- |
| Anesthesiology ICU | 9 |
| General ICU | 9 |
| Palliative Care Unit | 7 |
| Pandemic ICU | 5 |
| Neurology ICU | 5 |
| Coronary ICU | 4 |
| Internal Medicine ICU | 4 |
| Other wards* | 12 |
| **Total** | **55** |

*Other wards include General Surgery, Neurology ward, Orthopedics, Chest Diseases, Physical Therapy and Rehabilitation, Stroke Unit, Day Clinic, and other single-case units.
